# Age Determination by Back Length for African Savanna Elephants: Extending Age Assessment Techniques for Aerial-Based Surveys

**DOI:** 10.1371/journal.pone.0026614

**Published:** 2011-10-19

**Authors:** Morgan J. Trimble, Rudi J. van Aarde, Sam M. Ferreira, Camilla F. Nørgaard, Johan Fourie, Phyllis C. Lee, Cynthia J. Moss

**Affiliations:** 1 Conservation Ecology Research Unit, Department of Zoology and Entomology, University of Pretoria, Pretoria, South Africa; 2 Scientific Services, South African National Parks, Skukuza, South Africa; 3 Amboseli Trust for Elephants, Nairobi, Kenya; 4 Behaviour and Evolution Research Group, School of Natural Sciences, University of Stirling, Stirling, United Kingdom; University of California, Berkeley, United States of America

## Abstract

Determining the age of individuals in a population can lead to a better understanding of population dynamics through age structure analysis and estimation of age-specific fecundity and survival rates. Shoulder height has been used to accurately assign age to free-ranging African savanna elephants. However, back length may provide an analog measurable in aerial-based surveys. We assessed the relationship between back length and age for known-age elephants in Amboseli National Park, Kenya, and Addo Elephant National Park, South Africa. We also compared age- and sex-specific back lengths between these populations and compared adult female back lengths across 11 widely dispersed populations in five African countries. Sex-specific Von Bertalanffy growth curves provided a good fit to the back length data of known-age individuals. Based on back length, accurate ages could be assigned relatively precisely for females up to 23 years of age and males up to 17. The female back length curve allowed more precise age assignment to older females than the curve for shoulder height does, probably because of divergence between the respective growth curves. However, this did not appear to be the case for males, but the sample of known-age males was limited to ≤27 years. Age- and sex-specific back lengths were similar in Amboseli National Park and Addo Elephant National Park. Furthermore, while adult female back lengths in the three Zambian populations were generally shorter than in other populations, back lengths in the remaining eight populations did not differ significantly, in support of claims that growth patterns of African savanna elephants are similar over wide geographic regions. Thus, the growth curves presented here should allow researchers to use aerial-based surveys to assign ages to elephants with greater precision than previously possible and, therefore, to estimate population variables.

## Introduction

The response of vertebrate populations to environmental perturbations or to conservation based management actions is best assessed by measures of demographic variables including age structures, estimates of age-specific fecundity and survival, and derived intrinsic population growth rates [1]–[4]. This is true for Africa’s savanna elephants *Loxodonta africana* (see [5]–[7]), which are of special interest to many conservationists due to rapid population growth in some areas and steep population declines in others. Constructing age structures and estimating demographic parameters for elephants, as for other species, depends on reliable age assignment to individuals within the population [8].

Elephant shoulder height has frequently been used to assign ages to free-ranging elephants, and many shoulder height growth curves have been published (see [9] for a review) based on curves calibrated to elephants of known [9], [10] or estimated [11] ages. Digital photogrammetry techniques described by Shrader *et al.* [12] provide an accurate and efficient method to measure height and, thus, to estimate ages of free ranging elephants. Based on information gathered in this way for a sample of elephant herds in a population, demographic parameters can be estimated [13] and used to better understand the relationships between environmental variation, management interventions, and elephant population dynamics [5]–[7].

However, measuring shoulder heights to estimate age limits researchers to conducting ground-based surveys. This method is problematic where road access is limited, encounter rate for elephants is low, or vegetation impedes visibility, as is frequently the case. Even under good conditions for ground-based surveys, finding and measuring a sufficient sample of elephants can be time consuming. By contrast, aerial-based surveys are much faster and reduce the risk of inadvertently sampling a herd more than once.

Currently, the lack of a known growth curve for a variable that can be measured from above limits aerial-based sampling. The back length of elephants may provide such a variable. Like elephant shoulder height, back length continues to grow into adulthood and, perhaps, until death [14], [15]. Laws [15] used fixed-height flights and aerial photographs to derive back length measurements for elephants. He compared these to an age/length key developed from culled elephants in Murchison Falls National Park, Uganda and estimated the age and sex composition for each 10 cm interval in back length. However, precision was limited to 5-year age classes, and accuracy was unknown because the age/length key was derived from ages estimated from tooth replacement and wear patterns rather than known ages [15]. Croze [16] refined the technique to measure elephant back lengths from variable-height aerial surveys. However, the refined technique relied on a shoulder height growth curve to assign age, assuming any discrepancy between growth in height and back length was negligible, and involved calculating length relative to mean length of the largest individuals rather than actual length [16]. Consequently, these methods were problematic to implement [17] and relied on growth curves for which accuracy and precision were unknown [16], [17]. More recently, Shrader *et al.* [7] used a linear model to convert back lengths measured in aerial surveys to shoulder heights for age assignment. However, this model assumes an exact correlation and thus may introduce considerable errors to age assignment (in addition to the known uncertainty in the relationship between shoulder height and age) [9].

A growth curve with explicitly described errors that relates back length to age for known-age elephants would alleviate these problems and allow efficient age assignment to elephants via aerial survey. Therefore, we evaluated growth in back length for known-age African savanna elephants in Amboseli National Park, Kenya, and Addo Elephant National Park, South Africa, to provide parameter estimates for sex-specific Von Bertalanffy growth curves [18]. We also compared age- and sex-specific back lengths between Amboseli National Park and Addo Elephant National Park and compared adult female back lengths across 11 populations in Africa. We found support for the claim that African savanna elephant growth patterns are generally similar over broad geographical regions [9].

## Methods

### Ethics statement

Fieldwork was carried out in September 2003 in Addo Elephant National Park in South Africa (permission granted in a memorandum of understanding between the Conservation Ecology Research Unit and South African National Parks) and October 2004 in Amboseli National Park in Kenya (permission granted to the Amboseli Trust for Elephants by the Kenya National Council for Science and Technology, research permit number NCST/PRI/12/1/BS-011/04).

We adapted the digital photogrammetric techniques for shoulder height measurement described by Shrader *et al.* [12] to conduct ground-based measurements of back lengths of elephants in Amboseli National Park, Kenya, and Addo Elephant National Park, South Africa, where the year of birth is known for elephants born since 1970 [19] and 1976 [20] respectively. We measured back length as the distance of a line parallel to the ground between the point where the top of the ears meet the head and where the tail meets the body. These end points are easily recognizable from the air, and a line parallel to the ground ensures that equivalent values would be measured from the air or the ground (Fig. S1), provided that care is taken regarding position and angle of the camera relative to subjects (see later). During ground surveys, we could only measure back length of individuals that stood perpendicular to the camera. We measured the back length of 126 known-age elephants in Amboseli and 34 in Addo. We measured an additional 6 females in Addo and 12 in Amboseli that were born prior to 1970 and 1976 respectively, but for which approximate ages had been estimated with a reasonable degree of certainty in long-term studies [19], [20]. Fieldwork was carried out in September 2003 in Addo and October 2004 in Amboseli, and identification of elephants was determined by personnel who could identify individuals or by comparisons with reference photographs.

We fitted growth curves separately for back lengths of male and female elephants of known age and also for female elephants including older individuals with estimated ages. We used a Von Bertalanffy curve described by the equation *l_i_* =  *l_b_*+ (*l_∞_*−*l_b_*)(1−e^−*kt*^) where *l_i_* is back length, *l_b_* is back length at birth, *l_∞_* is asymptotic back length, *k* is a rate constant, and *t* is age [18]; this form of the equation limits the lower bounds to size at birth rather than conception (see [9]). We then compared our fitted growth curves for back length to published curves for shoulder height [9].

Given the fitted estimates and SEs of *l_b_*, *l_∞_*, and *k* for known-age males and known-age females, we used Monte Carlo simulations to assess uncertainty in age assignment from back length. Starting at 100 cm, we assigned age 1000 times to each back length in 1 cm increments while allowing the parameter estimates of the Von Bertalanffy growth curve to vary according to their SEs. Based on these simulated age estimates, we calculated the mean, standard deviation, and 95% confidence intervals of age for each 1 cm increment, stopping at a maximum back length where fewer than 100 of the 1000 trials returned values.

To assess whether growth is similar across populations, we used paired t-tests to compare age-specific mean shoulder heights separately for male and female elephants in Amboseli and Addo. We also compared back lengths of mature adult female elephants in 11 populations across five countries (Botswana, Mozambique, Namibia, South Africa, and Zambia) where we measured back lengths from a helicopter during dry seasons between 2004 and 2009. We used a photogrammetric technique [12] which we adapted for measuring back length from the air. We used a helicopter in which one person sat in the front seat to obtain digital images with a single lens reflex digital camera of sufficient resolution (see [12]) fitted with a lens of known focal length with low lens distortion, and a second person sat in the back seat on the same side with a laser rangefinder. For each herd sampled, we identified a “focal elephant” that had a distinguishing feature that would be recognizable later in images (e.g. a broken tusk, distinct mud splotch). The camera-observer, rangefinder-observer, and pilot worked together to simultaneously take an image of and a distance measure with the rangefinder to the focal elephant. The distance to the focal elephant was typically between 20 and 70 m, and digital photogrammetry is precise and accurate up to at least 120 m [12]. The photograph was taken such that the back length line of the focal elephant was perpendicular to the camera axis; however, it was not necessary to take a truly vertical aerial photograph (i.e. where tilt angle, the angle between the camera axis and nadir (plumb line from the camera to the ground), is zero). The image and distance measure were later used to derive back length in cm based on pixel counts (Fig. S1) and camera-specific calibration formulae developed to relate pixels to cm given the lens’ focal length and distance between camera and elephant [12].

To measure each elephant individually in this manner requires too much costly flying time, so to estimate back lengths of other individuals within each herd, we made a series of images per herd showing various elephants in the same frame as the focal elephant (Fig. S1). Two aspects of scale variation in aerial photography are relevant here: relief, or elevation of points above ground level, and camera tilt angle. In a true vertical photograph, scale is constant at a given height above ground, but points at different heights undergo relief displacement [21]. The magnitude of displacement on the photograph is equal to r * h/H where *r* is radial distance from the principal point of the photograph, and *h* is height of an object with respect to the ground, and *H* is distance from the camera to the ground. This presents a problem for measuring back lengths from aerial photos because for each elephant, the height of the point on the head and the tail differ, and small and large elephants differ in height. Relief displacement introduces errors to back length measures that increase with radial distance and are most severe for elephants facing directly towards or away from the principal point.

Croze [16] suggested a rough correction for relief displacement based on a standard ratio between true back length and rise between tail and head height (as measured for a sample of elephants on the ground); distance of each elephant from the principal point in the photograph; and whether each elephant is facing towards, away from, or side on to the principal point. While the correction accounts, roughly, for difference in height of points on each elephant, it does not take into account differences in small and large elephants and assumes the ground is exactly level. Because we were interested in ratios between individuals, we opted not to correct for relief displacement error, and we needed only to ensure that errors were similar between individuals to a reasonable margin. Thus, we restricted the individuals we measured in each photograph to ensure that relief displacement errors in back length measures did not differ by more than ∼2%. For example, in vertical photographs taken at 100 m elevation, we only measured individuals within approximately five body lengths of the principal point or for individuals away from the principal point that were within five body lengths of one another and were facing the same radial direction with respect to the principal point (Fig. S1).

While we attempted to ensure that photographs were truly vertical (or close enough to approximate vertical with tilt angle <5° [16], [21]), many photographs were visibly tilted. Tilt, in conjunction with relief, affects scale, *S*, at each point according to the formula *S* =  ((*f*/cos(*t*)) − (*y*' * sin(*t*)))/(*H*−*h*) where *f* is focal length, *t* is tilt angle, *y*' is photo-distance along the direction of tilt from the origin at the photo nadir, *H* is distance from camera to ground level, and *h* is height of the point above the ground [21]. While it is possible to correct measurements for tilt, the tilt angle was generally unknown. However, scale is constant in tilted photographs along lines perpendicular to the direction of tilt at constant height. The direction of tilt is generally obvious through visual inspection of photographs. Therefore, we once again restricted the individuals we measured in tilted photographs to ensure that appropriate scale was maintained and measurement errors minimized. We measured elephants standing side on (perpendicular to the optical axis) at an approximately equal distance from the photo nadir. What was deemed “approximately equal distance” to minimize error depended on the estimated degree of tilt and focal length, and we facilitated decisions on acceptable distance by dividing photographs into horizontal fractions, e.g. thirds or sixths. For example, when focal length was 70 mm and tilt was ≤10°, we could reliably measure elephants spread over one-third of the photograph (Fig. S1). Because the elephants were standing side on to the camera and radial distance was relatively similar, relief displacement issues were also minimized [16].

For a given photograph, we then estimated back length *l_i_* for each individual that met the measurement restrictions according to the formula *l_i_* =  *p_i_* * *l_f_*/*p_f_* where *p_i_* is the back length in pixels of the individual whose back length is to be estimated, and *l_f_* and *p_f_* are, respectively, the photogrammetrically measured back length in cm and back length in pixels of the focal individual. Often, it was necessary to use a series of photographs to get acceptable measurements for as many individuals within a herd as possible. We distinguished mature adult female elephants as those associating with two or more calves because females old enough to have two calves should be nearing the asymptote for growth [9]. We used ANOVA to compare back lengths among populations.

## Results

**Figure 1:**
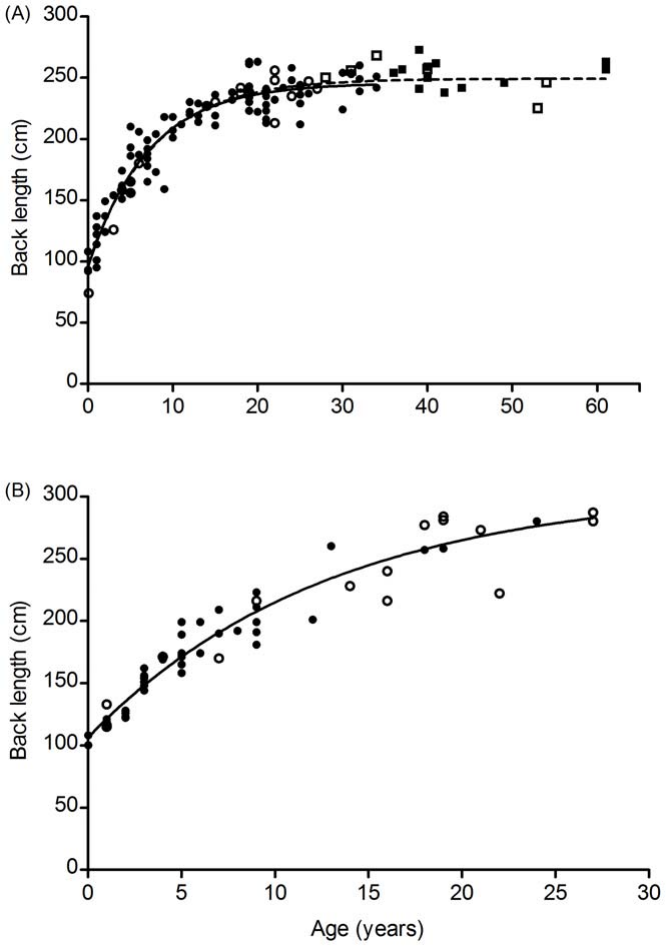
Elephant back length growth curves. Von Bertalanffy growth curves fitted to back length measurements of elephants in Amboseli National Park (closed symbols) and Addo Elephant National Park (open symbols). Two curves were fit for females (a); one (solid line) included only known-age elephants (circle symbols), and the other (dashed line) included an additional 18 individuals (square symbols) for which age was estimated. One curve was fit for known-age males (b).

We constructed growth curves based on back lengths of 56 male elephants and 104 female elephants of known age and for 122 females including 18 of estimated age (Fig. 1, Table 1). Including the 18 females that were born prior to the onset of birth record keeping, but for which age was estimated, did not substantially affect parameter estimates of the growth curve. The mean back length of those 18 females was 252.5 cm (SD = 11.6 cm). This mean is slightly higher than the asymptotic back length of 245.5 cm predicted by the growth curve for females of known age, yet within the 95% confidence limits of *l_∞_* estimated from the dataset including the older, estimated-age individuals, i.e. 244.4–253.8 cm. The asymptotic back length predicted for males was greater than we observed in our sample of males, which was limited to ≤27 years of age.

**Table 1:** Parameters of growth curves, *li* = *l_b_*+ (*l_∞_*−*l_b_*)(1−e^−*kt*^), where *l_i_* is back length, *l_b_* is back length at birth, *l_∞_* is asymptotic back length, *k* is a rate constant, and *t* is age in years, fit to back length data from known-age male elephants, known-age females, and known- and estimated-age females from Amboseli National Park and Addo Elephant National Park.

	Known-age males (n = 56)		Known-age females (n = 104)		Known- & estimated-age females (n = 122)	
	Estimate (SE)	95% CL	Estimate (SE)	95% CL	Estimate (SE)	95% CL
*l_b_*	106.2 (5.419)	95.34–117.1	95.34 (4.681)	86.04–104.6	96.87 (4.473)	88.02–105.7
*l_∞_*	307.0 (15.33)	276.3–337.8	245.5 (2.981)	239.6–251.5	249.1 (2.377)	244.4–253.8
*k*	0.0780 (0.0130)	0.0519–0.1040	0.1408 (0.0112)	0.1186–0.1631	0.1317 (0.0091)	0.1137–0.1497
*R^2^*	0.93		0.91		0.92	

**Figure 2:**
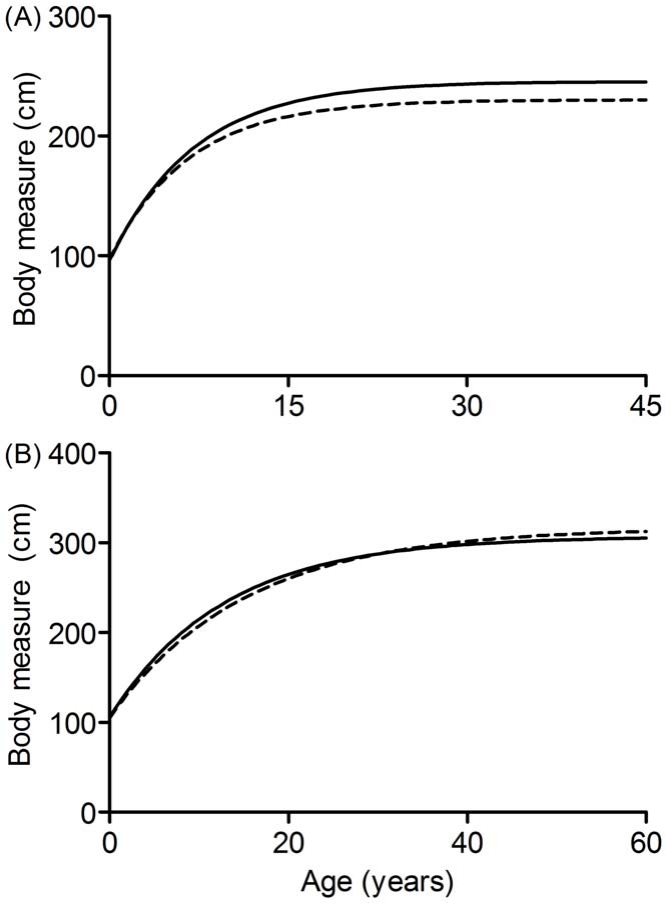
Back length and shoulder height growth. Growth curves for female (a) and male (b) back lengths (solid lines) and shoulder heights (dashed lines). Shoulder height growth curves are based on parameterization of Von Bertalanaffy curves (females: *h_i_* = 96.9+ (230.2−96.9)(1−e^−0.150*t*^); males: *h_i_* = 105.4+ (316.6−105.4)(1−e^−0.066*t*^) where *h_i_* is measured shoulder height and *t* is age in years) published by Shrader *et al.* [9], and back length curves follow the parameterization for known-age males and females (Table 1).

We compared sex-specific shoulder height growth curves [9] with back length curves. This comparison showed divergent growth patterns for shoulder height and back length for females but more similar growth curves for the two in males (Fig. 2). After similar initial growth for female shoulder height and back length, back length increased more than shoulder height to reach a higher asymptote, i.e. 245.5 cm for back length versus 230.2 cm for shoulder height.

**Figure 3:**
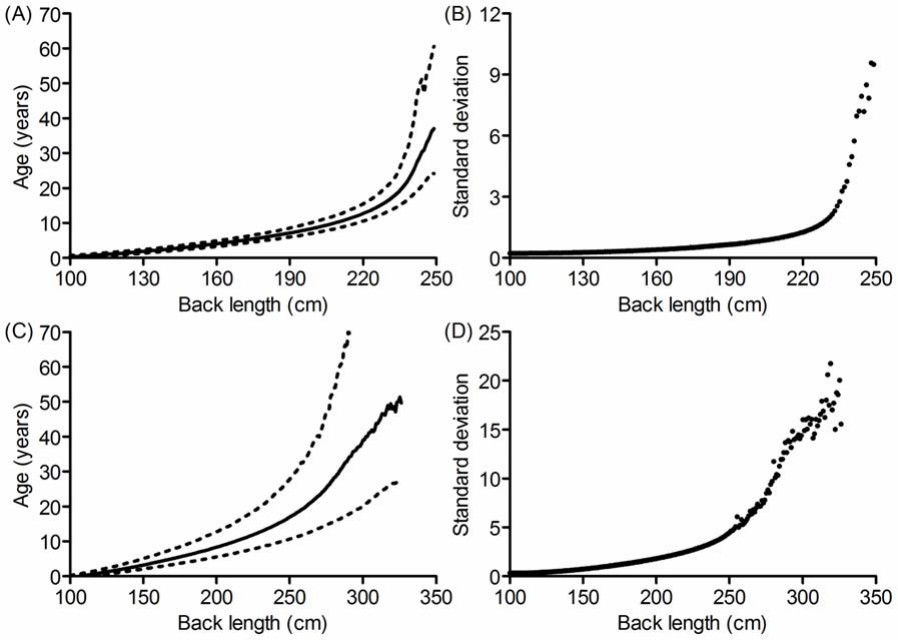
Monte Carlo precision assessment. Mean age and 95% confidence intervals for females (a) and males (c) generated by 1000 Monte Carlo simulations per 1 cm increment in back length to assign age based on parameter estimates and SEs for growth curves fitted to known-age males and females. Precision was greater for females though standard deviation increased with age for both females (b) and males (d).

Monte Carlo simulations demonstrated that precision in age assignment decreased as back length increased (Fig. 3, Table 2). Age estimates for females were relatively precise (SD ≤5 years) up to 240 cm but less so for males, and SD exceeded 5 years at a back length of 254 cm.

**Table 2:** Mean age and 95% confidence intervals (LCL = lower confidence limit, UCL = upper confidence limit) for subset of back lengths constrained by *l_b_* and *l_∞_* calculated from Monte Carlo simulations.

	Females			Males		
Back length (cm)	Age (years)	95% LCL	95% UCL	Age (years)	95% LCL	95% UCL
100	0	0	0			
105	0	0	0			
110	0	0	1	0	0	0
115	0	0	1	0	0	1
120	1	0	1	0	0	1
125	1	1	2	1	0	2
130	1	1	2	1	0	2
135	2	1	2	2	1	3
140	2	1	3	2	1	3
145	2	2	3	2	1	4
150	3	2	3	3	2	5
155	3	2	4	3	2	5
160	4	3	4	4	2	6
165	4	3	5	4	3	7
170	4	4	5	5	3	7
175	5	4	6	5	3	8
180	5	4	7	6	4	9
185	6	5	7	6	4	9
190	7	5	8	7	4	10
195	7	6	9	7	5	11
200	8	7	10	8	5	12
205	9	7	11	8	5	13
210	10	8	12	9	6	14
215	11	9	13	10	6	16
220	12	10	15	11	7	17
225	14	11	17	11	7	18
230	16	13	20	12	8	19
235	19	14	25	13	8	21
240	24	17	36	14	9	23
245	30	21	47	15	10	25
250				16	10	27
255				18	11	30
260				19	11	33
265				21	12	37
270				23	13	39
275				25	14	47
280				28	15	53
285				30	16	60
290				33	17	70
295				36	18	73
300				39	20	80
305				41	21	82

**Figure 4:**
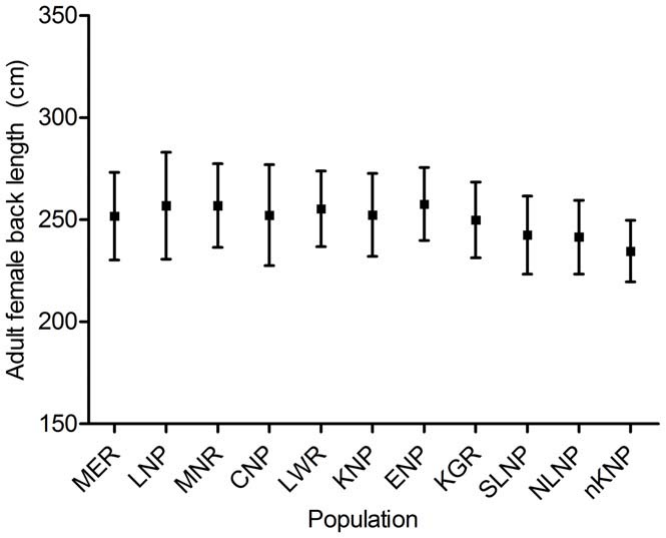
Back length across populations. Mean back length and SD of adult females (those with at least two calves) in 11 savanna elephant populations. Population abbreviations, country, survey year, and sample size as follows: MER—Maputo Elephant Reserve, Mozambique, 2009, 25; LNP—Limpopo National Park, Mozambique, 2009, 19; MWR—Moremi Wildlife Reserve, Botswana, 2008, 37; CNP—Chobe National Park, Botswana, 2008, 83; LWR—Linyanti Wildlife Reserve, Botswana 2006, 66, KNP—Kruger National Park, South Africa, 2009, 205; ENP—Etosha National Park, Namibia, 2004, 34; KGR—Khaudum Game Reserve, Namibia, 2004, 51; SLNP—South Luangwa National Park, Zambia, 2004, 90; NLNP—North Luangwa National Park, Zambia, 2004, 97; nKNP—northern Kafue National Park, Zambia, 2004, 84.

Age-specific mean back length did not differ significantly between Addo Elephant National Park in South Africa and Amboseli National Park in Kenya for females (paired t_13_ = 1.04, mean of differences = 4.17 cm longer in Amboseli, P = 0.32) or males (paired t_4_ = 0.28, mean of differences = 2.54 cm longer in Addo, P = 0.79); however, for males, there were few paired values to compare (Fig. 1). Mean back length of adult females (those with at least 2 calves) ranged from 234.6 cm in North Kafue to 257.6 cm in Etosha (Fig. 4) and did differ significantly across the 11 populations (F_10,780_ = 9.89, P<0.01). The significant difference was driven by the three populations in Zambia, South Luangwa National Park, North Luangwa National Park, and the northern part of Kafue National Park, which had shorter mean back lengths than any other populations. When these were excluded, mean back length did not differ significantly among the remaining 8 populations (F_7,512_ = 0.85, P = 0.54).

## Discussion

Our study builds on previous attempts to use back length to assign ages to elephants [15]–[17] by providing sex-specific Von Bertalanffy growth curves based on back lengths of known-age elephants. Von Bertalanffy growth curves have been used to model growth on age of elephant shoulder height [9], [10], [22], [23], weight [22], [23], and foot length [10]. Von Bertalanffy curves also provide a good fit for back length data and are an important addition to previously developed shoulder height curves (e.g. [9]) because, unlike shoulder height, back length can be measured in aerial-based surveys. These surveys, while costly, can be more practical than ground-based surveys, especially when a large number of elephants needs to be measured in a relatively short time, e.g. when sampling a population to estimate demographic variables [13]. However, researchers seeking to apply this method should consider and control for the effects of tilt and relief displacement in aerial photography.

To create back length growth curves, we relied on measuring known-age elephants in Amboseli and Addo, two widely separated populations subject to long-term study [19], [20]. We were limited to a ground-based survey to enable identification of individuals in these populations. However, measuring back length from the ground with digital photogrammetry requires the elephants to be standing on level ground, perpendicular to the camera. This limited our sample size, and in particular, we lacked samples from older males. The back lengths of known-age males measured in our study did not reach the asymptotic length predicted by the male growth curve, i.e. 307 cm. Thus, including older males may affect the parameterization of the model, which may result in greater distinction between shoulder height and back length growth curves. Nonetheless, the growth curve suggested that males may not reach asymptotic length within their lifetime. They were predicted to reach 306 cm at nearly 70 years of age, in agreement with assertions that back length increases until death [14], [15]. We were able to measure older females for which age was estimated rather than known, but including these had little effect on the female growth curve. Female back length was within 1 cm of the predicted asymptotic length at 35 years of age and thereafter, slowly increased towards the asymptote of 245.5 cm.

The leveling of the growth curve for females limited relatively precise age assignment (SD ≤5 years) to 240 cm, or 23 years of age. By contrast, the standard deviation of age predicted based on female shoulder height growth curves exceeds 5 years at a height of 215 cm, equivalent to 15 years of age [9]. Thus, back length may allow more precise age assignment to older females than shoulder height does, probably because back length continues to grow for longer than shoulder height [15] as demonstrated by a comparison of the two growth curves. However, back length did not allow for more precision in age assignment to males than did shoulder height. Shoulder height up to 290 cm (31 years) predicts male age with SD ≤5 years [9], while the back length growth curve presented here lost precision at 254 cm, only 17 years of age. However, the back length growth curves relied on less than half the sample size available for Shrader *et al.* 's [9] shoulder height curves. Despite identical *R^2^* values for shoulder height and back length growth curves, parameter estimates for the shoulder height curves have substantially lower SEs [9]. Therefore, further refining the back length growth curves by increasing the sample size may reduce the SEs of the parameter estimates and allow greater precision in age assignment, particularly for males.

We found similar age- and sex-specific back lengths in Addo Elephant National Park in South Africa and Amboseli National Park in Kenya; these populations are isolated from one another and geographically separated by at least 6000 km. Despite the geographic separation, trajectories of height growth are also similar between these two populations [9]. Additionally, we found that adult female back length was similar across eight populations. These findings agree with previous studies [9], [11] that suggest growth patterns of savanna elephants are generally similar over much of Africa, and thus, that growth curves generated in one population can be used to estimate age elsewhere. While shorter back lengths in the Zambian populations identified here could indicate deviant growth patterns, it may be more likely that larger animals have simply succumbed to poaching, a well-documented problem in Zambia [24], [25]. More research is needed in this regard. Furthermore, though growth patterns appear to be generally similar, local ecological factors could influence growth of individual elephants with consequences for age estimation. For example, severe environmental constraints such as extreme drought or continued exposure to energy limitation might affect elephant growth, especially early in life [26], as is seen in other mammals [27]. Ongoing research on populations where individuals and ages are known and environmental conditions are monitored could quantify such effects [26]. If changing environmental conditions do substantially affect growth, the growth curves presented here will overestimate precision and may need refinement.

In conclusion, the sex-specific growth curves presented here will allow researchers to use aerial-based surveys to assign relatively accurate ages with associated estimates of precision to African savanna elephants in age classes relevant to demographic assessments for estimating population variables. For example, assigning ages to individuals in mother–calf associations allows estimation of reproductive variables, i.e. age at first calving and calving interval, which can be used to estimate population growth [13]. Furthermore, deviations from predicted age structures can be used to assess responses to environmental variation and management [5], [6]. For such applications of age data, we recommend that researchers retain estimates of uncertainty in age assignment to a specific year class in further analytical modeling and/or minimize uncertainty by grouping into multi-year age classes.

Previous attempts to measure back length in aerial surveys to assign ages to elephants suffered from several shortcomings including the need for fixed-height flights and use of a radio-altimeter and reliance on unsubstantiated growth curves [15]–[17] or linear conversion from back length to shoulder height [7], which incorporated additional, un-modeled errors to age assignment. However, combining the known-age back length growth curves presented here with recently developed digital photogrammetric techniques [12] should allow more flexible, reliable, and efficient aerial-surveys of African savanna elephants that can be incorporated into recurrent population monitoring routines.

## Supporting Information

Figure S1 **Measuring back length.** Lines demonstrating how back length is measured in pixels for a single elephant on the ground (a), a single elephant from the air (b), and a herd of elephants from the air (c & d). We measured back length between end points where the top of the ears meet the head and where the tail attaches to the body. To minimize errors due to effects of relief and tilt displacement, we restricted the elephants we measured in tilted photographs (c) to those standing perpendicular to the optical axis at similar distance from the nadir along the axis of tilt. In vertical photographs (d), we restricted the elephants measured to those standing near the principal point or away from the principal point but near each other and facing the same radial direction.
(JPG)
