# Supplementary figures and images for: Age Determination by Back Length for African Savanna Elephants: Extending Age Assessment Techniques for Aerial-Based Surveys
Source: PLoS One. 2011 Oct 19;6(10):e26614. doi: 10.1371/journal.pone.0026614 (PMC3197571; doi:10.1371/journal.pone.0026614)

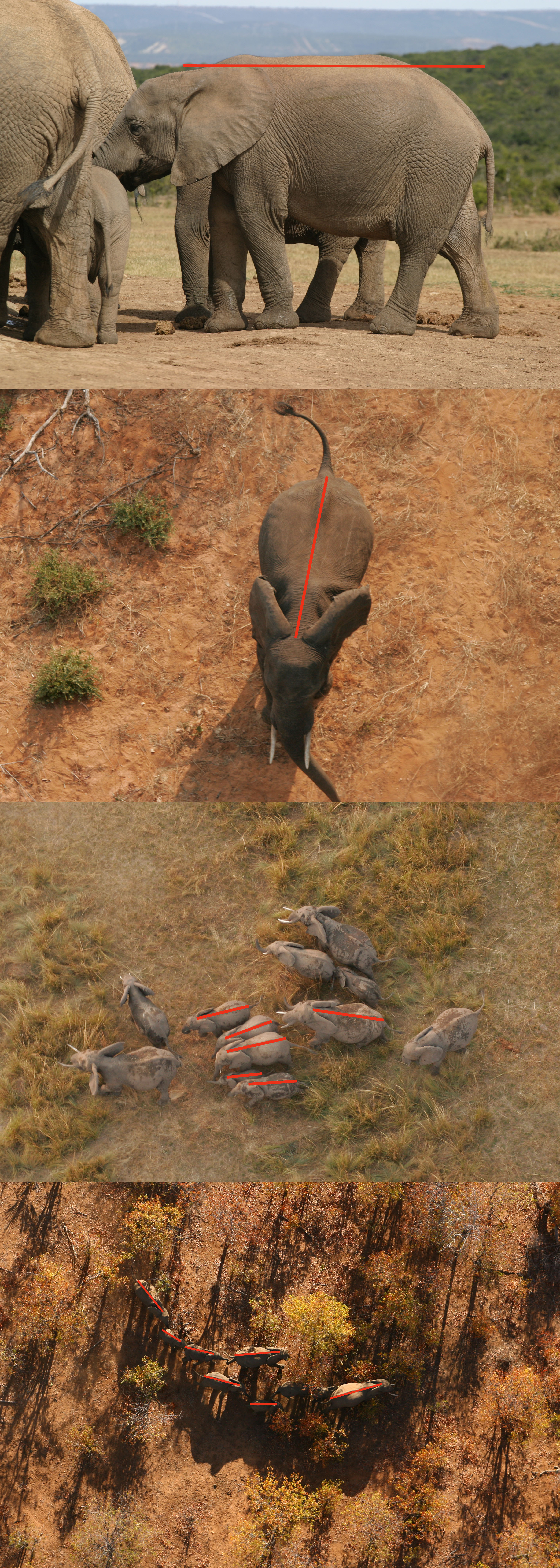

Supplement: Figure S1 — Measuring back length. Lines demonstrating how back length is measured in pixels for a single elephant on the ground (a), a single elephant from the air (b), and a herd of elephants from the air (c & d). We measured back length between end points where the top of the ears meet the head and where the tail attaches to the body. To minimize errors due to effects of relief and tilt displacement, we restricted the elephants we measured in tilted photographs (c) to those standing perpendicular to the optical axis at similar distance from the nadir along the axis of tilt. In vertical photographs (d), we restricted the elephants measured to those standing near the principal point or away from the principal point but near each other and facing the same radial direction. (JPG) [file pone.0026614.s001.jpg]
